# A Systematic Literature Review of Efficiency Measurement in Nursing Homes

**DOI:** 10.3390/ijerph16122186

**Published:** 2019-06-20

**Authors:** Alice Tran, Kim-Huong Nguyen, Len Gray, Tracy Comans

**Affiliations:** Centre for Health Services Research, Faculty of Medicine, the University of Queensland, Building 33, Princess Alexandra Hospital Campus, Woolloongabba QLD 4102, Australia; kim.h.nguyen@uq.edu.au (K.-H.N.); len.gray@uq.edu.au (L.G.); t.comans@uq.edu.au (T.C.)

**Keywords:** aged care, nursing home, residential, retirement, long-term care, assisted living, efficiency, inefficiency, productivity, performance, literature review

## Abstract

*Background*: As our population ages at an increasing rate, the demand for nursing homes is rising. The challenge will be for nursing homes to maintain efficiency with limited resources while not compromising quality. This study aimed to review the nursing home efficiency literature to survey the application of efficiency methods and the measurements of inputs, outputs, facility characteristics and operational environment, with a special focus on quality measurement. *Methods:* We systematically searched three databases for eligible studies published in English between January 1995 and December 2018, supplemented by an exhaustive search of reference lists of included studies. The studies included were available in full text, their units of analysis were nursing homes, and the analytical methods and efficiency scores were clearly reported. *Results:* We identified 39 studies meeting the inclusion criteria, of which 31 accounted for quality measures. Standard efficiency measurement techniques, data envelopment analysis and stochastic frontier method, and their specifications (orientation, returns to scale, functional forms and error term assumptions) were adequately applied. Measurements of inputs, outputs and control variables were relatively homogenous while quality measures varied. Notably, most studies did not include all three quality dimensions (structure, process and outcome). One study claimed to include quality of life; however, it was not a well-validated and widely used measure. The impacts of quality on efficiency estimates were mixed. The effect of quality on the ranking of nursing home efficiency was rarely reported. *Conclusions:* When measuring nursing home efficiency, it is crucial to adjust for quality of care and resident’s quality of life because the ultimate output of nursing homes is quality-adjusted days living in the facility. Quality measures should reflect their multidimensionality and not be limited to quality of throughput (health-related events). More reliable estimation of nursing home efficiencies will require better routine data collection within the facility, where well-validated quality measures become an essential part of the minimum data requirement. It is also recommended that different efficiency methods and assumptions, and alternative measures of inputs, outputs and quality, are used for sensitivity analyses to ensure the robustness and validity of findings.

## 1. Introduction

For the first time in history, the majority of the global population can expect to live over 60, contributing to a strong increase in demand for aged-care services and placements in nursing homes [1]. Direct challenges for nursing homes are a steady increase in operational costs and higher expectations of service quality [2,3]. To maintain business vitality, the only way to deal with these challenges is to improve productivity and efficiency through service and process innovations [3]. In fact, improving nursing home efficiency while maintaining quality is the key for sustainable development of aged-care settings [3,4,5].

Productivity and efficiency are two related yet distinct concepts [6]. While the productivity of an organisation (e.g., a nursing home) refers to the ratio of outputs (products or services) to the inputs (resources), efficiency refers to the extent to which the facility achieves the highest feasible productivity [6,7,8]. It is expressed as the proportion of the nursing home productivity to the maximum productivity (of similar organisations). As such, efficiency is measured on a scale of 0 to 1 (or 0% to 100%), where a value of 1 indicates the facility has the highest possible productivity, and a value less than 1 indicates a lower productivity level (relative to the best), i.e., having some inefficiency [6,7,8]. Outputs might include (number of) residents, services provided, and resident days. Typical inputs that a nursing home uses include the (number of) rooms (and/or beds), full-time-equivalent staff hours (by categories), administration and supplies/consumables. The efficiency of a nursing home is affected by many factors, including resident dependency level, staffing mix, facility location, management and ownership structure, and operational objectives. Measuring efficiency and understanding the sources of inefficiencies (if any) can help facilities identify how well they are operating in relation to similar organisations and determine where to focus to improve their productivity [6].

There are two main methods of measuring efficiency: parametric and non-parametric approaches (detailed information can be found in Appendix 1 in the Supplementary Material). The parametric approach involves the estimation of a production function using econometric techniques [7]. Stochastic frontier analysis is the dominant parametric method, which conceptualises the production process (goods or services) through an additive mathematical operation of a monotonous and concave function, an (in)efficiency component, and an error term that captures the statistical noise or measurement errors [7]. The non-parametric approach uses linear programming to establish the production frontier, from which productivities and efficiencies are calculated [7]. Data envelopment analysis is the predominant non-parametric method. Depending on the data used, both methods can be used to calculate technical efficiency, scale efficiency, cost efficiency, and allocative efficiency. When only input and output quantities are used, technical and scale efficiencies can be calculated. When price information is also available for individual input and outputs, one can calculate all four efficiency indicators (see, for instance, [6,7]).

The choice of efficiency methods and models, the quantity and price measure of inputs and outputs, and other control variables can result in different efficiency scores, and subsequently (but not less important) the ranking of relative performance (of production units). Efficiency ranking provides a summary of best performers (highest efficiency scores, often equal to one) and the worst performers (lowest efficiency scores). Consumers and managers of a particular nursing home can use the ranking to understand their relative position compared to other nursing homes with similar characteristics (such as size, location, ownership and operational model), from which sources of inefficiency may be identified and potential changes could be introduced to improve efficiency. The choices of efficiency methods, models, and measures were investigated in detail in the hospital context using a meta-regression analysis approach [9]. They found that estimated mean efficiency scores were affected by sample size (lowering scores, with diminishing effect), number of variables included in the model (increasing scores, with diminishing effect), flexible functional form, and variable returns to scale (increasing scores). The choices of parameterisation, orientations and error distribution did not have statistically significant impacts on efficiency score estimates. However, they did not investigate the impact of quality measurement on efficiency.

In aged care, both efficiency and quality are central concerns for policymakers. It is undesirable to improve efficiency by compromising quality of services because better health outcomes and improved wellbeing of the older adults are the ultimate goals of a good aged care system. Therefore, measuring efficiency without proper adjustments for quality differences between facilities will not provide accurate information about their relative performances, thereby not facilitating true improvements [10]. For instance, if the number of bed-days, without adjusting for quality of care provided for residents, is considered a primary output of the nursing home, a facility could reduce the number of staff per resident, or employ unskilled staff, to reduce costs while keeping the same number of bed-days. While both a low staff–resident ratio and unskilled staff can compromise the quality of services, the facility appears more efficient if quality is not incorporated into efficiency estimates.

The quality of care is *multidimensional*. Based on Donabedian’s conceptual framework [11,12], which remains the foundation of quality assessment today [13], care quality entails the three dimensions of structure, process and outcome. “Structure” is defined as the settings, qualifications of providers, and administrative systems through which care takes place. “Process” refers to the components of care delivered, including both the health professional’s activities (e.g., nursing care, diagnosis, treatment or patient education) and the health care consumer’s activities in seeking care and carrying it out (e.g., choice of treatment). “Outcome” refers to recovery, restoration of function and/or survival of the service recipients. Measuring quality with a high level of precision, and identifying the determinants of quality, however, remain major challenges in both practice and research.

From the lens of *healthy ageing* in older adults, quality in nursing homes involves both quality of care and quality of life. Both are important parts of clinical governance, accreditation and, increasingly, consumers’ preferences [14]. While quality of care refers to the structure and process of care, quality of life is a broader measure that refers to outcomes contributing to a patient’s wellbeing and/or overall health. There is a growing consensus that *healthy ageing* and *a good health and aged care system* are more than just the absence of disease/disability or the increased length of life [15,16,17,18]; they are about improving or maintaining health-related quality of life and wellbeing within the society’s limited economic resources [1].

This study was motivated by this context. To our knowledge, this is the first systematic review of productivity and efficiency analyses of nursing homes with a particular focus on the quality aspects. We aim to answer the following questions:

1. Which methods have been used to measure productivity and efficiency of nursing homes?
2. How did these studies measure quantities and prices of inputs and outputs, and how did they account for the different characteristics of nursing home operations?
3. Did these studies adjust for quality of care and/or quality of life (of the residents)? If they did, how was quality measured? Did they appropriately reflect the outputs that a nursing home aims to produce?

## 2. Methods

The systematic review followed the Preferred Reporting Items for Systematic Reviews and Meta-Analysis (PRISMA statement) [19].

### 2.1. Search Strategies

We searched three electronic databases using keyword searches within titles and abstracts of articles: ECONLIT, PubMed (Medline), and Web of Science. Our search terms string for all three databases were: (efficienc* OR productiv* OR performance OR inefficien*) AND (“data envelopment” OR DEA OR stochastic OR SFA OR parametric OR econometric* OR non-parametric OR nonparametric OR malmquist) AND (aged OR ageing OR aging OR “aged care” OR residential OR retirement OR “nursing home” or “long term care” OR “assisted living”). The lateral searching methods are provided in Appendix 2 in the Supplementary Material.

We included studies in which (i) the decision-making unit (or unit of analysis) was nursing homes (including assisted living facilities, home for the aged, residential aged care facilities, long-term care facilities), and (ii) the analytical methods and efficiency scores were clearly reported. The analytical methods include, but are not limited to, linear programming, data envelopment analysis, stochastic frontier analysis, least-square estimation, and total factor productivity analyses. Studies measuring efficiency of aged care services, programs, and/or the whole aged care system were excluded. The search was limited to peer-reviewed articles, doctoral dissertations and conference proceedings published between January 1995 and December 2018, written in English, and available in full text.

### 2.2. Study Selection

We imported studies identified through database searches into reference management software (Zotero). Hand-searching was also applied to screen reference lists of included studies to identify other relevant articles that may have been missed in the indexing process or were not indexed in the search databases. Duplicate records and non-English language articles were removed. One reviewer (A.T.) scanned titles and abstracts, and identified articles required for full-text evaluation. Full-text articles were reviewed if the available information indicated that the study met the inclusion criteria or if there was doubt based on the title and/or abstract. The final inclusion of full-text studies was determined by majority consensus through discussion of all reviewers (A.T., K-H.N., and T.C.).

### 2.3. Data Extraction

Key details of studies were extracted, including title, authors, reference/source, country of study, year of publication, type of aged care facility, sample size, and methods used to estimate efficiency. Detailed information on efficiency models was also extracted and organised by efficiency measures (technical, scale, cost and/or allocative efficiency), orientation and returns to scale, methods, model specifications, number of estimation stages, measures of quantities, and prices of inputs, outputs and control variables. Quality measures were examined closely and classified as adjustments for inputs, outputs or as control variables. We also reported mean efficiency scores and the impacts of quality measures on the efficiency estimates.

## 3. Results

### 3.1. Summary of Included Studies

**Figure 1:**
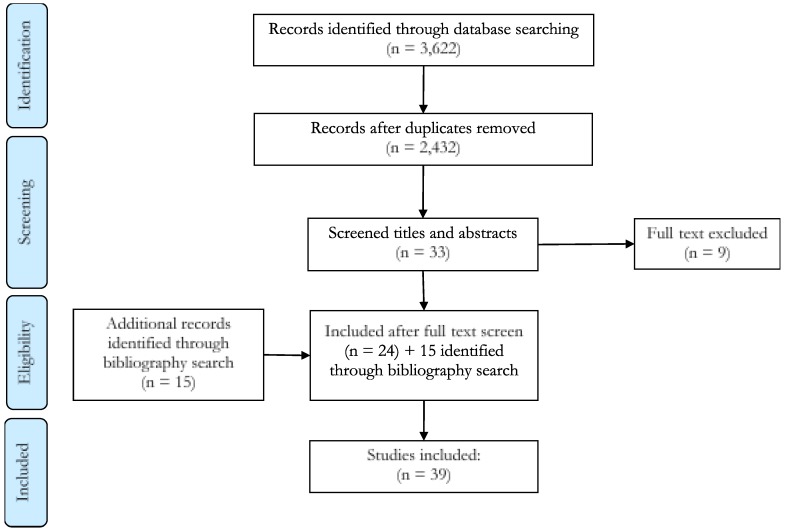
PRISMA (Preferred Reporting Items for Systematic Reviews and Meta-Analyses) flow diagram of the search and screening process for the current systematic review.

A total of 3622 studies were identified through the database search process, of which 24 met the inclusion criteria for the review. The text and reference lists of these 24 documents were manually searched, resulting in 15 additional records being identified as eligible for inclusion, giving a total of 39 studies (Figure 1). Studies included 20 from the USA, six from Switzerland, five from Finland, three from Taiwan, two from France and one each from Canada, Ireland and Italy. Out of 39 studies, 31 accounted for quality measures in their models and 35 reported efficiency scores. There are 129 models involved in the 39 included studies; 102 models reported efficiency scores. A detailed description of the included studies can be found in Appendix 3 in the Supplementary Material.

The mean efficiency score of the 102 models that reported efficiency score was 0.75. Only 14 models reported all three statistical values of efficiency scores: mean (0.83), median (0.88), and standard deviation (0.156). Twenty-three models reported either median or standard deviation. About 20% of all the studies found a mean efficiency score higher than 0.9. Studies with highest mean efficiency score were from the US [2,20], Switzerland [21,22,23], and Taiwan [24]. The prominent characteristics of the nursing homes with higher efficiency scores were: for-profit (operational incentive), high occupancy rate (operational factor), and competitive market (environmental factor). Of all the studies, 18 included at least one of these characteristics, and 14 adjusted for quality [2,24,25,26,27,28,29,30,31,32,33,34,35,36]. These 14 studies each included a relatively large number of variables, ranging from 7 to 25 (mean = 15, SD = 5), and the majority of them used data envelopment analysis.

### 3.2. Estimation Methods and Model Specifications

From the 39 included studies, we extracted 129 estimated models. As shown in Table 1, the majority of the studies estimated technical efficiency of nursing homes (27/39), compared to cost efficiency (13/39), owing to the unavailability of reliable price information for inputs and outputs in nursing homes. The majority of the studies (33/39) used an input orientation (including those that estimated cost efficiency). One study used output orientation; four studies used profit orientation and three studies did not clearly state the orientation assumption. While input orientation assumes that facilities minimise the input or cost to achieve the desirable level of outputs (i.e., their service demand is given), output orientation assumes facilities maximise outputs/revenue from their fixed resources (i.e., they provide as much as they can, given the resources they have). The number of studies reporting efficiency under constant returns-to-scale (CRS) and variable returns-to-scale (VRS) are 17/39 and 28/39, respectively. It should be noted that under the CRS assumption both the input-oriented and output-oriented efficiency will be the same while these two estimates are likely to be different under VRS, which is a more realistic assumption.

More than a half of the studies used data envelopment analysis (DEA) (22/39), followed by those using stochastic frontier analysis (SFA) (12/39). The majority of the DEA studies consisted of two stages (14/22). Of the 22 DEA studies, 19 used input orientation and one used output orientation. Most of DEA studies (68%) used CRS, and 59% used VRS. *(Note that these percentages do not add up to 100% because some studies might use both CRS and VRS assumptions)*.

The majority of SFA studies followed a one-stage process (8/12). Ten studies estimated input-orientated technical efficiency, and nine estimated cost efficiencies. Two studies did not report an orientation assumption. The majority of SFA studies (10/12) assumed VRS, either by explicit reporting or by the nature of the estimate functions used. Two SFA studies assumed CRS as they used a linear production function [32,37].

### 3.3. Input and Output Measures

**Figure 2:**
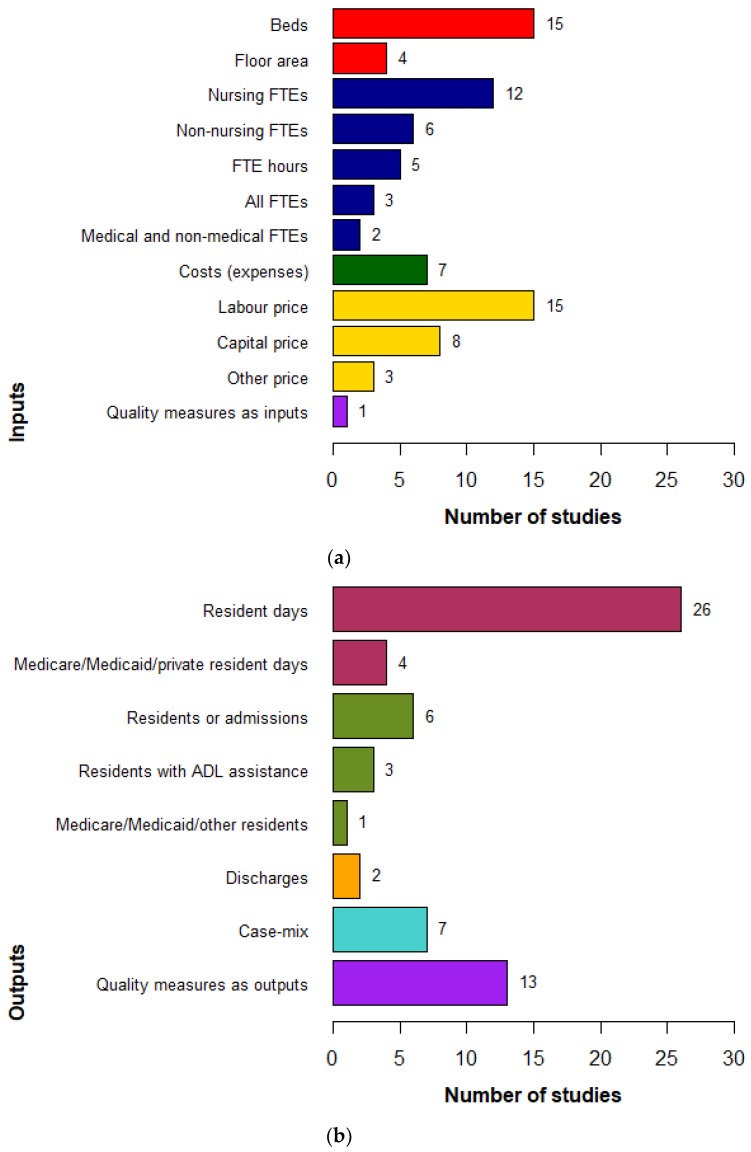
Distribution of input and output measures in nursing home efficiency literature: (**a**) distribution of input measures; (**b**) distribution of input measures.

The summary of input and output measures reported in these studies is shown in Figure 2 and Table 2.

The most frequently used measure of physical capital (input) was the number of beds. As opposed to the labour input—which is categorised into groups, as described below—beds were not split into categories, such as high dependency and low dependency beds. The most common measures of labour (input) were staff expenditure ($ salary) and/or the number of full-time equivalent staff, which was broken down by skill categories (e.g., registered nurses, licensed practical nurses, and nursing assistants). In studies where input prices were available, price (or cost) items follow the corresponding quantities of outputs and inputs. They often included labour prices, capital cost [21,22,23,38,39] and material expenditure [21].

The main output measures in the literature were “number of residential days”, with or without casemix adjustment, and “number of residents” (Figure 2b and Table 2). Where a casemix system (e.g., RUG-III) was available, its weights could be used to aggregate all episodes of resident services into a single output of resident days [32,40,41,42,43]. Alternatively, casemix was used to create output categories, e.g., casemix severity of activities of daily living [44,45,46,47]. When a casemix system was not available, outputs were measured by number of resident days or number of residents by different categories, for example: reimbursement types [20,26,27,48] or resident care types, such as high or low care [35,36]. While it was common practice to use casemix-adjusted outputs, no studies used quality-adjusted measures of output.

### 3.4. Control Variables

Control variables in the efficiency analysis literature consist of all measures that are neither quantities nor prices of inputs and outputs but might affect the production technologies (i.e., the position of the production frontier) or the production process of individual units (i.e., the productivity). If the former position was assumed (we labelled it Approach 1, shown in Table 3), control variables were included in the production (or cost) function, following all the input and output variables. These studies always used stochastic frontier method or other parametric methods. If the latter was used, control variables either entered the (in)efficiency component (using stochastic frontier method—we labelled this Approach 2a) or were included as covariates in the second-stage regression (Approach 2b).

In this literature, we observed all three approaches. Of 29 studies that used control variables, 16 studies incorporated control variables directly into the estimated equations; 14 studies (both SFA and DEA) used control variables in a second-stage estimation; and two SFA studies incorporated control variables in the (in)efficiency terms. While these assumptions often had noticeable impacts on efficiency estimates, the direction of impacts are mixed, as shown in Table 3.

Control variables can be roughly categorised into two groups: facility characteristics and environmental factors. Facility characteristics included for-profit status (12/39), chain affiliation (11/39), ownership (10/39), and occupancy rate (10/39). It appeared that large for-profit facilities with higher occupancy rate had higher productivity, and thus gave the impression of being more efficient than their counterparts. Environmental factors often covered location (e.g., rural vs. metropolitan/urban) (10/39) and market competition, measured by the Herfindahl–Hirschman index (6/39). Market competition may have an impact on efficiency by motivating the facility to operate efficiently to maintain their competitiveness. The Herfindahl–Hirschman index is a common measure of market concentration: the sum of squared market share of each firm within the market. These two factors—location and market competition—had mixed impacts on efficiency estimates (see details in Table 3).

Some studies also explored the impact of changes in reimbursement policy [23,36,53]. Dormont and Martin (2012) [53] found that pricing reform—from cost reimbursement to a prospective payment system based on the degree of dependency of residents—would improve efficiency only if minimum standards of staff ratios were applied. This reform does not reduce inefficiencies, but reduces quality. Farsi et al. (2008) [23] measured the positive effect of reimbursement per resident on total cost (effect on efficiency estimate was not reported). Meanwhile, Zhang et al. (2008) [36] showed the significantly negative impact of Medicare prospective payment system changes on efficiency.

### 3.5. Quality Measures

The review found that 31/39 (74%) studies included at least one quality measure in their efficiency analyses. Nineteen studies (of the 31) incorporated only one quality dimension: either structure (8/19) or outcome (11/19). Nine studies included both structure and outcome dimensions, and one study covered all three dimensions of quality, including a quality-of-life measure [26]. In this study, quality of life was defined as the “degree of involvement in the provision of organised groups for its residents and their families” [26] (p. 45).

The majority of studies incorporated quality measures as control variables (19/29) or as a measure of output (13/29). Similar quality measures were used as outputs in some studies—e.g., “prevalence of falls” [2,25,28,34]—but as control variables as in others [42]. One study used quality measures (% non-ambulatory, % not self-feeding) as inputs [54]. Table 4 and Table 5 list all quality measures used and how they were incorporated into the efficiency analyses. None of the studies used quality-adjusted resident days or residents as outputs.

In ten studies (see Table 5), the quality measures were entered directly into the production or cost function; ten used quality measures in the second stage analysis; two incorporated them into the efficiency term when estimating a cost function using the stochastic frontier method [28,53].

Of those reporting the impact of quality on efficiency, the impacts were found to be mixed. Four studies found no impact [22,31,35,43]. Four studies found that accounting for quality increased efficiency estimates of all facilities [25,29,39,53]; of which two directly incorporated quality measures into their SFA function (Approach 1) [29,39] and one incorporated quality measures in error terms [53] and one used quality measures (number of falls and times the residents used emergency services) as outputs in their DEA model [25]. Four studies showed a negative impact of quality on the efficiency estimates [23,36,42,52]; of which three used DEA, either in the second stage analysis [42,52] or as an output [36], and one incorporated quality measures directly into their SFA function [23].

Two studies investigated the association between quality measures and other characteristics of nursing homes. Knox et al. [30,31] found no significant difference in quality among chain-affiliated versus independent, or for-profit versus not-for-profit, facilities. The former found that number of beds and resident dependency level (measured by casemix) were negatively correlated with quality. Lastly, urban facilities appeared to have lower quality than their rural counterparts.

## 4. Discussion

In this study, we conducted a systematic review of productivity and efficiency analyses of nursing homes. We wanted to understand the variation of efficiency estimates by different modelling choices and measurements of inputs, outputs and other characteristics and attributes of nursing homes, especially the quality domain. To our best knowledge, this is the first comprehensive review of this literature.

The analyses of nursing home efficiencies is concentrated in a handful of countries (the US, European countries and Taiwan), most of them are OECD nations. Within the search strategies of this review, however, no study was identified in other OECD countries such as Australia, New Zealand or Japan. This might due to a low demand for such analysis and/or the unavailability of (sufficient) nursing home data for researchers to conduct the analysis. In Australia, for example, while nursing homes are subject to regular accreditation and quality assessment, most meet the minimum required standard [56]. There exists no comprehensive “ranking” of nursing home performance that older individuals and their families can review when making the decision to move to a nursing home. Admission is often negotiated privately, based on location and ability of individuals and their families to pay (using both private and Commonwealth funded budgets). Additionally, large nursing homes have often enjoyed a local monopoly, resulting in very little incentive to perform better than average. Aged care data, including resident assessments of care needs and level of care packages (e.g., by Aged Care Funding Instrument (ACFI) score), utilisation of primary health services (e.g., general practitioners and specialist services) and medications, and hospitals admission (including emergency) are regularly collected and reported to the Commonwealth. However, these data are not easily accessible, due to both privacy regulations and access/linkage cost. With the shift to high-value care, the introduction of consumer-directed care in the aged care market, and rising concerns with quality of care in nursing homes, the need for performance analyses and ranking might become more acute.

This review found that most studies in the literature were standard applications of efficiency analysis methods (e.g., data envelopment or stochastic frontier analyses) and the estimation assumptions (e.g., returns to scale, and orientation). When comparing the effect of returns-to-scale assumptions on efficiency estimates (while keeping others equal), some studies [24,52] found that imposing a constant-returns-to-scale assumption on the model reduced the efficiency score. This is similar to the findings observed in hospital literature [57]. Interestingly, included studies with large sample sizes (*n* > 1000) in this review tend to have very low efficiency scores (<0.4) [34,36,53]; which differs from hospital literature where they observed little change in efficiency estimates when sample size was large [9].

The measurement of inputs and outputs, as well as facility characteristics and environmental factors, are relatively homogenous across studies. Most studies used number of beds [25,27,44,48,49], number of full-time nurses [33,35,40,44,54], or labour price [21,23,38,43,55] as inputs; and used number of resident days [2,32,33,40,55], number of residents [24,25,45,46,47], or case-mix index [20,28,30,31,51] as outputs. The use of inputs in nursing home efficiency measurement is similar to the input measures in hospital literature; i.e., number of beds, labour inputs such as doctors or nurses, or input prices [9,58]. Most frequently used outputs are different between the two settings as they capture different services that are provided. While commonly used outputs representing residential aged care services found in this review are number of days living in nursing homes or number of residents, the common output measures in hospital efficiency studies are inpatients, outpatients, episodes of care, or emergency department presentations [9,58].

While the impacts of control variables, including quality measures, on efficiency estimates were mixed across studies, it appeared that large for-profit facilities with higher occupancy rates had higher efficiency scores than their counterparts [30,34,35]. This might be the effect of scope and scale economies and profit-oriented business models, which are often found in other efficiency literature regarding hospital settings [59,60]. Regarding the density of competition (environmental factor), the findings of this review are similar to the recent findings of hospital efficiency studies, where some observed a positive relationship between the competition level and the efficiency score [60,61], and others observed a negative effect [62] or statistical non-significance [63]. Hence, it is recommended to have the correct incentives, such as competition based on value not just service, while considering other sources of revenue to avoid the undesirable outcomes of the incentive payment systems [62].

The definitions and measurements of quality in nursing homes varied, both for inputs and outputs, and factors influencing the “service production” in the facility. The impacts of quality measure on efficiency estimates were mixed. Notably, there is only one study [26] (p. 45) that accounted for quality of life of residents (“degree of involvement in the provision of organised groups for its residents and their families”). This, however, is not a standardised quality-of-life measure that has been used widely or well-validated. These findings highlight a critical aspect of efficiency measurement in nursing home literature: the need for more precise measurements of quality for both outputs and inputs. It is imperative to account for quality when analysing performance and such quality measures should be standardised. Non-standardised quality measures lead to mixed results of impact of quality on efficiency. A nursing home that increases the number of occupied beds or employs low-skill staff will inevitably reduce operation costs while producing the same number resident bed-days. If the bed-days are not quality adjusted, it is likely that the low-cost nursing homes that produce low-quality services to residents will appear more efficient than their counterparts that invested in facility and staff skill to provide the best possible care to their residents. The efficiency estimates and ranking in such a case are invalid (i.e., failing to distinguish low- and high-quality services) and might lead to misleading conclusions about what an efficient model of care should look like. This means we would fail to distinguish low- and high-value care.

Nursing homes provide a “home” for older adults where they can maintain their health and wellbeing in the last years of their lives. This means the ultimate output should capture both “life length” and “quality of life”, or quality-adjusted days living in the nursing home. While counting length of life (e.g., days in a nursing home [29,33,40,44,55,64]) and severity/dependency (e.g., using ADL dependency, casemix, frailty index [2,21,27,28,34]) is relatively straightforward, it is unclear what the most reliable measures are to capture quality of service and/or quality of life of residents. This is what we found in this review: “numbers of resident days” [29,33,40,44,55,64] or “number of residents” [24,25,45,46,47] were commonly used, but the quality adjustment varied widely from percentage of adverse events (e.g., pressure ulcer [2,43,54], fall [25,28,34], infection [2,28,34], and medication error [26]) to some measures of health decline (e.g., ALD deterioration [2,28,34]). Potential reasons for these wide variations were that quality measures were not routinely collected as part of the administration process and/or these measures were not made available for researchers [65].

Faced with imperfect measures of quality for the final output, studies often opted to use quality-adjusted throughputs and inputs as its approximation. This reflects the belief that high-quality services result in better quality of life of residents. This assumption is reasonable because all the services provided in the facility, from personal care to activities and health services are throughputs (intermediate outputs) for the final output (residents’ health outcomes and wellbeing). However, the quality relationship is not a direct one-to-one for two reasons. First, the definitions and measurements of service quality are not perfect; similar to measuring quality of outputs/outcomes. For instance, a high nurse–resident ratio was used extensively as an input-related quality measure in this literature, but this might only capture the ability to reduce adverse events (e.g., pressure ulcer) rather than provide personal care. Second, a good score in some quality domains (e.g., rate of falls, or use of antibiotics) might be driven by activities that do not necessarily lead to health improvement (and sometimes safety) and enjoyment (e.g., restraint to prevent falls and behaviour problems, use of antibiotics to stop infections spreading, or limited physical activities for residents with low mobility to prevent falls or fractures). Lastly, these quality measures share a common property, in that they focus on measuring health-related events rather than “wellbeing” or “quality of life”, which are self-evaluations of life satisfaction [66].

Quality instruments to measure quality of life and quality of care for nursing homes are well-developed and/or widely used. However, such quality-of-life instruments have not been used in any of the efficiency studies found in this review; examples for these instruments are ASCOT [67,68], interRAi QoL-LTCF [69,70], and WHOQOL-Old [71]. Regarding quality-of-care instruments, in Australia, quality measures have been discussed in the Australia’s Single Quality Framework [14] and National Aged Care Quality Index Program [72]. In the US, the one-to-five star rating guideline for nursing homes are based on three quality domains: health inspections, staffing levels, and quality measures based on minimum data base and claims [73]. Internationally, instruments like interRAI for long-term care facilities (LTCFs) quality index [74] have been developed and validated in Canada, New Zealand, Europe and the US. However, the use of these frameworks and instruments is not always a strict requirement in routine data administration in nursing homes. Therefore, they are not consistently available for measuring quality and, ultimately, relative performance of facilities. One potential solution to improve the reliability of efficiency measurements in nursing homes is to establish a minimum list of quality indices that cover the quality of care domains and quality of life of residents, and ensure that these are regularly collected as part of nursing administration processes.

Our study highlights the variations of efficiency estimates with regard to methods, model assumptions and measurements of inputs, outputs and control variables. While data envelopment analysis has been the most popular method (as it can easily accommodate multiple outputs and inputs), its drawbacks lie in its deterministic nature (i.e., no statistical error term) and inability to account for the potential effects of other variables on the production process (i.e., the position of the production frontier). Stochastic frontier analysis can deal with these limitations but is more limited in the inclusions of multiple inputs and outputs, unless a distance function approach is applied [8]. It is therefore important that researchers use both methods and conduct extensive sensitivity analyses around the assumptions of functional form and statistical error, and, ideally, different definitions and measurement of inputs, outputs and quality. For instance, efficiency scores and ranking resulting from estimation with and without quality measurement can be compared and contrasted to highlight the domains where quality can be improved. This will allow for an in-depth understanding of the source of true inefficiency versus statistical and measurement errors. Only when this practice is applied consistently and widely in efficiency analyses of nursing homes do performance scores and ranking become relevant to business managers and policy makers in the aged care sector.

Our study has several limitations around the search strategies. First, we only used three databases—ECONLIT, PubMed (Medline), and Web of Science—for the published literature. It is possible that some relevant articles would not be discoverable by these databases. However, these databases are the three most comprehensive in economics, medicine and health, and we have complemented the formal search with additional hand searches of the reference lists in included studies. We believed that while this is not perfect, relevant studies were adequately found. Second, we did not include the grey literature because there were no studies found in either the databases or the hand searches. There may be government reports or working papers produced by nursing home authorities or health/aged care departments that were not reached in this literature review. Third, we only included studies that have full text in English due to the time and resource limitations for translations of non-English publications, if they exist.

## 5. Conclusions

When measuring nursing home efficiency, it is crucial to adjust for quality of care and resident’s quality of life because the ultimate output of nursing homes is quality-adjusted days living in the facility. Quality measures should reflect their multidimensionality and not be limited to quality of throughput (health-related events). More reliable estimation of nursing home efficiencies will require better routine data collection within the facility, where well-validated quality measures become an essential part of the minimum data requirement. It is also recommended that different efficiency methods and assumptions, and alternative measures of inputs, outputs and quality, are used for sensitivity analyses to ensure the robustness and validity of findings.

## Figures and Tables

**Table 1:** Summary of methods used in included studies.

	DEA (22/39)		SFA (12/39)		Others (7/39)		Total (39/39)	
**Number of stages** *(sum up to 100%)*								
1 stage	32%	(7/22)	67%	(8/12)	57%	(4/7)	43%	(17/39)
2 stages	64%	(14 /22)	33%	(4/12)	14%	(1/7)	49%	(19/39)
3 stages	4%	(01/22)	0%	(0/12)	29%	(2/7)	8%	(3/39)
**Return to scale** *(not sum up to 100%)*								
CRS	68%	(15/22)	17%	(2/12)	0%	(0/7)	44%	(17/39)
VRS	59%	(13/22)	83%	(10/12)	100%	(7/7)	72%	(28/39)
N/A	0%	(0/22)	0%	(0/12)	0%	(0/7)	0%	(0/39)
**Orientation** *(not sum up to 100%)*								
ITE	86%	(19/22)	83%	(10/12)	71%	(5/7)	85%	(33/39)
OTE	5%	(1/22)	0%	(0/12)	0%	(0/7)	3%	(1/39)
Profit orientation	5%	(1/22)	0%	(0/12)	43%	(3/7)	10%	(4/39)
N/A	5%	(1/22)	17%	(2/12)	14%	(1/7)	8%	(3/39)
**Frontier** *(sum up to 100%)*								
Cost frontier	4%	(1/22)	75%	(9/12)	72%	(5/7)	36%	(14/39)
Production frontier	32%	(7/22)	25%	(3/12)	14%	(1/7)	26%	(10/39)
Others	64%	(14/22)	0%	(0/22)	14%	(1/7)	38%	(15/39)

DEA = data envelopment analysis; SFA = stochastic frontier analysis; CRS = constant returns to scale; VRS = variable returns to scale; ITE = input-oriented technical efficiency; OTE = output-oriented technical efficiency; N/A = not available. **Notes**: One-stage analysis: the determinants of efficiency are estimated simultaneously with the efficiency scores, by a direct inclusion of the determinants in the efficiency term in a stochastic frontier model; Two- or three-stage analyses: efficiency scores are first estimated (stage 1), then scores are then regressed against the determinants using methods such as ordinary least squares, Tobit or logistic regressions. In both types of analysis, the determinants often include quality measures, case-mix indices, facility characteristics (e.g., ownership status, for-profit status, occupancy rate), and environmental factors (e.g., location, competitiveness).

**Table 2:** Summary of variables used in included studies.

Input	Output	Control
***Physical capital:*** No. of beds: 38% (15/39) Floor area: 10% (4/39) ***Human capital:*** Nursing FTEs: 31% (12/39) Non-nursing FTEs: 15% (6/39) Medical and non-medical FTEs: 5% (2/39) FTE hours: 13% (5/39) All FTEs: 8% (3/39) ***Input price:*** Labour price: 38% (15/39) Capital price: 21% (8/39) Other price: 8% (3/39) ***Other resource expenses:*** 18% (7/39) ***Quality measures:* 3% (1/39)** Outcome: 3% (1/39) (% non-ambulatory residents; % ambulatory and self-feeding residents)	***Residential services produced:*** (Un)adjusted resident days: 67% (26/39) Medicare/Medicaid/private resident days: 10% (4/39) Residents or admissions: 15% (6/39) Medicare/Medicaid/other residents: 3% (1/39) Discharges: 5% (2/39) Casemix: 15% (6/39) ***Consequence of receiving the services:*** Residents with ADL assistance: 8% (3/39) ***Quality measures:* 33% (13/39)** Structure: 15% (6/39) Process: 3% (1/39) Outcome: 31% (12/39)	***Structural characteristics:*** Ownership: 26% (10/39) For-profit status: 31% (12/39) Chain affiliation: 28% (11/39) Supervision: 3% (1/39) Medicare (Medicaid) admissions: 15% (6/39) Reimbursement per resident: 3% (1/39) Beds: 18% (7/39) Occupancy rate: 26% (10/39) Facility type: 23% (9/39) Ward’s specification: 5% (2/39) Others: 10% (4/39) ***Environmental factors:*** Location or urban/rural: 26% (10/39) HHI index: 15% (6/39) No. of agencies in area: 3% (1/39) Area average income: 8% (3/39) Wage index: 3% (1/39) % population aged 84+: 3% (1/39) County occupancy rate: 3% (1/39) ***Other control factors:*** Reimbursement (dummy): 8% (3/39) Policy change: 10% (4/39) Time trend: 28% (11/39) CM - age: 5% (2/39); discharge rate: 3% (1/39); - acuity/ADL index: 21% (8/39); others: 5% (2/39) ***Quality measures:* 51% (20/39)** Structure: 38% (15/39) Process: 0% (0/39) Outcome: 23% (9/39)

FTE = full-time equivalent; ADL = activity of daily living; HHI = Herfindahl–Hirschman index; CM = casemix; No. = Number of.

**Table 3:** Impacts on efficiency and types of main control variables.

	Impact on Efficiency	Approach 1	Approach 2a	Approach 2b
**Facility characteristics**				
For-profit	Positive (*n* = 9)	[30,35,37,49]	[28]	[2,34,36,50]
	Negative (*n* = 2)	[29]		[27]
Private-owned	Positive (*n* = 3)			[25,36,51]
	Negative (*n* = 3)	[29]		[27,52]
	Mixed impacts depends on models (*n* = 2)	[31]	[53]	
	Insignificant or no impact (*n* = 3)	[21,38]		[24]
Chain affiliation	Positive (*n* = 3)	[49]		[34,36]
	Negative (*n* = 2)		[28]	[50]
	Mixed impacts depends on models (*n* = 3)	[30,31]		[36]
	Insignificant or no impact (*n* = 3)	[29]		[2,27]
Occupancy rate	Positive (*n* = 8)	[30,31]		[24,25,32,34,35,48]
	Negative (*n* = 1)			[33]
	Mixed impacts depends on models (*n* = 1)			[36]
Size (number of beds)	Positive (*n* = 5)	[29]		[34,35,36,52]
	Negative (*n* = 1)			[24]
	Insignificant or no impact (*n* = 1)			[51]
**Environmental factors**				
Competition (measured by HHI index)	Positive (*n* = 2)			[27,36]
	Insignificant or no impact (*n* = 3)	[29,35]		[24]
Urban	Positive (*n* = 3)	[29,30,31]		
	Mixed impacts depends on models (*n* = 2)		[53]	[52]
	Insignificant or no impact (*n* = 2)	[49]		[48]

Notes: Approach 1: control variables were incorporated directly into estimated equations; Approach 2a: control variables were incorporated in the error terms, only applicable for studies using SFA; Approach 2b: control variables were incorporated in the second-stage analyses.

**Table 4:** Quality dimensions in the included studies, using the framework of structure, process, and outcome [11].

	Structure (19/39)	Process (1/39)	Outcome (20/39)
**Inputs** **(1/39)**			1. % Non-ambulatory: 3% (1/39) 2. % resident not self-feeding: 3% (1/39) *(These patient conditions as quality measures incorporated as inputs in Duffy et al.’s study* [54] *to reflect opportunity for patient co-production)*
**Outputs** **(13/39)**	1. Rating for health inspection (deficiencies): 10% (4/39) 2. FTEs contributing to QOC (e.g., RN) per resident: 3% (1/39) 3. FTEs contributing to QOL (e.g., activity professionals and staff) per resident (used as QOL measure): 3% (1/39) 4. Administrative service performance: 3% (1/39) 5. Life care performance: 3% (1/39) 6. Health care performance: 3% (1/39) 7. Extra nursing hours: 3% (1/39)	1. Degree of involvement in the provision of organized groups for its residents and their families QOL (used as QOL index): 3% (1/39)	1. % ADL decline: 8% (3/39) 2. % pressure ulcers: 21% (8/39) 3. % restraints: 18% (7/39) 4. % UTI: 8% (3/39) 5. % depression without treatment: 3% (1/39) 6. % pain: 8% (3/39) 7. % (no) falls: 10% (4/39) 8. Catheterisation: 15% (6/39) 9. % drug error: 3% (1/39) 10. Accident rate (or emergencies): 5% (2/39) 11. Out-of-pocket charges (as residential satisfaction—Willing to pay): 3% (1/39)
**Control variables** **(20/39)**	1. Staffing (RN hours/total nursing hours perresident day): 8% (3/39) 2. Nursing staff ratio (Nurses employed/nurses that should be employed according to the guidelines): 10% (4/39) 3. High quality facility (Care person per resident): 8% (3/39) 4. Nursing home rating: 8% (3/39) 5. Qualification of medical staff: 5% (2/39) 6. % RNs: 3% (1/39) 7. % rooms with own toilet: 3% (1/39) 8. % single rooms: 3% (1/39) 9. Average assistance time: 3% (1/39)		1. ADL severity level: 13% (5/39) 2. Acuity index: 3% (1/39) 3. Pressure sores: 10% (4/39) 4. Catheterisations: 8% (3/39) 5. Restraints: 10% (4/39) 6. Bedfast: 5% (2/39) 7. Unplanned weight change: 5% (2/39) 8. Depression: 8% (3/39) 9. Antipsychotic, anti-anxiety/hypnotic use: 8% (3/39) 10. Behavioural symptoms: 3% (1/39) 11. Cognitive impairment: 3% (1/39) 12. % use ≥ 9 medications: 3% (1/39) 13. Bowel incontinence: 3% (1/39) 14. Bladder incontinence: 3% (1/39) 15. % UTI: 3% (1/39) 16. % injury, fall, fracture: 3% (1/39) 17. Pneumococcal vaccination: 3% (1/39) 18. Influenza vaccination: 3% (1/39) 19. On pain management: 3% (1/39) 20. Adjusted mortality rate: 3% (1/39)

Abbreviations: FTE = full-time equivalent; QOC = quality of care; QOL = quality of life; RN = registered nurse; ADL = activity of daily living; UTI = Urinary tract infection.

**Table 5:** Incorporated types of quality indicators into control variables.

No.	Quality Measures			Approach 1	Approach 2a	Approach 2b
				(10/39)	(2/39)	(8/39)
	**Structure**					
1	Staffing (RN hours/total nursing hours per resident day)	8%	(3/39)	[23]	[28]	[2]
2	Nursing staff ratio (Nurses Employed/nurses that should be employed according to the guidelines)	10%	(4/39)	[21,22,39,55]		
3	High quality facility (Care person per resident)	8%	(3/39)	[23,38]	[53]	
4	Nursing home rating	10%	(4/39)	[28,30,31]		[33]
5	Qualification of medical staff	3%	(1/39)			[52], * Not as quality measure [25]
6	% RNs	3%	(1/39)			[42]
7	% rooms with own toilet	3%	(1/39)			
8	% single rooms	3%	(1/39)			
9	Average assistance time	3%	(1/39)	[38]		
	**Outcome**					
1	ADL severity level	15%	(6/39)	[21,22,39,55] * Not as quality measure [20,26,30,31]	* Not as quality measure [53]	[27,42] * Not as quality measure [34,35]
2	Acuity index	3%	(1/39)			[27]
3	Pressure sores	10%	(4/39)			[27,32,35,42]
4	Catheterisations	8%	(3/39)			[27,35,42]
5	Restraints	10%	(4/39)			[27,35,42,43]
6	Bedfast	5%	(2/39)			[27,42]
7	Unplanned weight change	5%	(2/39)			[27,42]
8	Depression	8%	(3/39)			[27,32,42]
9	Antipsychotic, anti-anxiety/hypnotic use	8%	(3/39)			[32,42,43]
10	Behavioural symptoms	3%	(1/39)			[42]
11	Cognitive impairment	3%	(1/39)			[42]
12	% use >= 9 medications	3%	(1/39)			[42]
13	Bowel incontinence	5%	(2/39)			[27,42]
14	Bladder incontinence	3%	(1/39)			[27]
15	% UTI	3%	(1/39)			[42]
16	% injury, fall, fracture	3%	(1/39)			[42]
17	Pneumococcal vaccination	3%	(1/39)			[27]
18	Influenza vaccination	3%	(1/39)			[27]
19	On pain management	3%	(1/39)			[27]
20	Adjusted mortality rate	3%	(1/39)	[29]		

Abbreviations: RN = registered nurse; ADL = activity of daily living; UTI = Urinary tract infection. **Notes**: Approach 1: control variables were incorporated directly into estimated equations; Approach 2a: control variables were incorporated in the error terms, only applicable for studies using SFA; Approach 2b: control variables were incorporated in the second-stage analyses.

## Supplementary Materials

The following appendices are available on Supplementary Material at https://www.mdpi.com/1660-4601/16/12/2186/s1, Appendix 1: Efficiency measurement, Appendix 2: Search terms and lateral searching methods, Appendix 3: Detailed description of the included studies.
