# Supplementary material for: A Systematic Literature Review of Efficiency Measurement in Nursing Homes
Source: Int J Environ Res Public Health. 2019 Jun 20;16(12):2186. doi: 10.3390/ijerph16122186 (PMC6616898; doi:10.3390/ijerph16122186)
Supplement: Supplementary file 1 [file ijerph-16-02186-s001.pdf]

Supplementary Material

Contents

Appendix 1 - Efficiency measurement 2
Appendix 2 - Search terms and lateral searching methods 5
Appendix 3 - Detailed description of the included studies 6
References 22

Appendix 1 - Efficiency measurement

All production requires the use of resources such as equipment and buildings (often referred to as capital), personnel (as labour), and land and raw materials. We can regard production as a process by which these resources are transformed into goods or services. Measures of efficiency can be defined as "ex post measures of how well firm managers have solved different optimisation problems" [1]. To measure how well a decision-making unit (DMU) perform in producing outputs (goods or services) from inputs (resources) and we need to know about their managerial behaviour (optimisation problems), for which the existing sets and functions has few implications for behaviour. For instance, revenue function does not mean that DMU managers will choose outputs in order to maximise revenues. Instead, different DMU managers tends to behave in different ways depending on what they can and cannot choose and on what they value. Some of the simplest optimisation problems that DMU managers face involve minimising inputs, maximising outputs, and/or maximising productivity [1].

Efficiency answers the question if any waste can be eliminated without worsening any inputs or outputs [2]. It is considered inefficient if the desired outcome can be achieved with less throughputs or the throughputs could produce more outcome desired.

Following are concepts of measuring efficiency which is also applied in health care:

Economic efficiency, or overall efficiency, refers to an economic state in which objectives are achieved in relation to the inputs (economic resources) used. It is estimated by the value of inputs employed and value of outputs delivered. Economic efficiency can be measured when price information is available and optimisation assumption—eg. cost minimisation, profit/revenue maximisation—is appropriate [3]. When the objective is revenue maximisation, a production function or output-oriented approach can be used to estimate revenue efficiency. When the cost minimisation is more appropriate, a cost function or input-oriented approach can be applied to measure cost-efficiency.

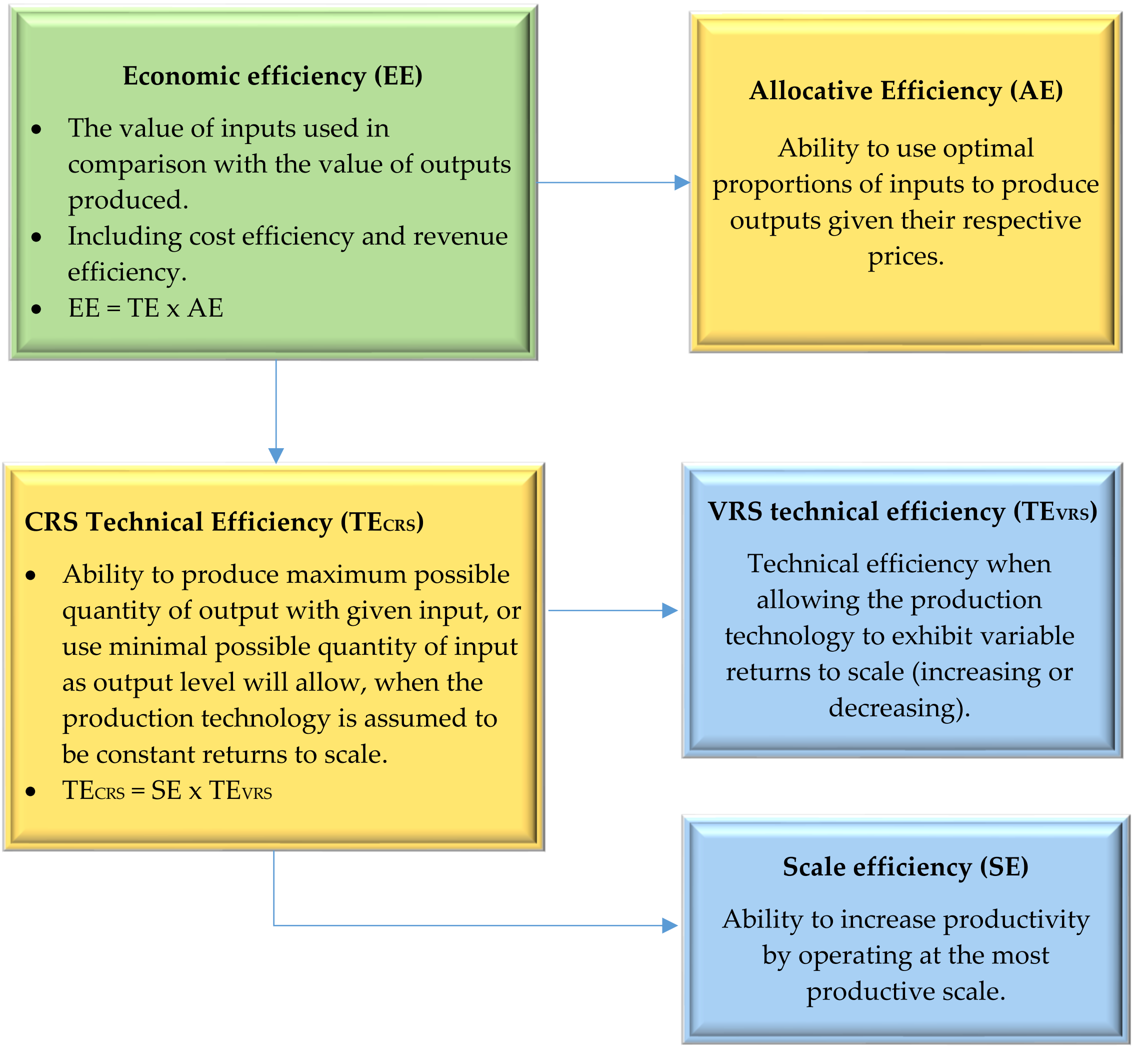

Technical efficiency refers to the measures of how well technologies are chosen and used [1]. It measures the ability of a DMU to avoid waste by minimising inputs as output level will allow or maximising outputs as input usage will allow. Technical efficiency can be categorised in terms of non-scale and scale effects. The former is considered as pure technical efficiency which technical efficiency under a variable return to scale (VRS) production technology. Scale efficiency measures the ability to eliminate waste by operating at the optimal productive scale. It is about operation size and how various sizes influence productivity and efficiency of the DMU. A DMU is referred to be at optimal scale only when it attains the highest possible productivity (ratio of output to input) with the available technology.

Allocative efficiency reflects the ability of a DMU to use their available inputs in optimal proportions given the available production technology and their respective prices. It is about

choosing between technically efficient combinations of inputs used to produce the maximum possible outputs.

Two major methods to measure efficiency are non-parametric and parametric methods. The non-parametric method is a piecewise-linear convex hull approach to frontier estimation originally proposed by Farrell [4], developed by Charnes et al. [5]; Banker et al. [6] and Fare et al. [7]. Data Envelopment Analysis (DEA), the predominant representative of non-parametric method, applies linear programming approach to estimate the production technology. DEA is often described as a non-parametric method as it does not involve any error terms. As such, it does not involve any assumptions about the functional form of the technology or the parameters (means, variances) of the distributions of those error terms. DEA requires assumptions regarding the regularity properties of the production frontier. For example, if the production possibilities set is not convex then the DEA model is known as a Free Disposal Hull model. DEA's assumption on functional form is that the cost or production frontier is locally linear.

The parametric method has stochastic frontier analysis (SFA) as the predominant representative. SFA involves the use of econometric methods to measure either primal or dual representations of the production technology. It was first developed simultaneously by Aigner et al. [8], Meeusen and Van den Broeck [9] and Battese and Corra [10]. Since then, SFA has evolved and become an increasingly popular method. SFA assumes the functional form of the frontier (e.g. translog or linear), the regularity properties of the frontier (e.g. monotonicity or concavity), and the distributions of error terms representing inefficiency and statistical noise (e.g. means or variances). The maximum likelihood method is usually used to estimate the unknown parameters of these functions and error distributions. The choice of functional representation is based on available data. For example, if only data on quantities of inputs and outputs are available, we can only estimate production frontiers, input and/or output distance functions. If we can only have access to the data on output quantities and input prices, we can only estimate cost frontiers.

Appendix 2 - Search terms and lateral searching methods

Base on terms related to “aged care facilities” (U.S. National Library of Medicine, https://meshb-prev.nlm.nih.gov/#/treeSearch), the type of facilities will be covered in our study are: Assisted living facilities, Home for the Aged, Nursing homes. Studies presented measurement approaches of aged care facility efficiency, which include, but are not limited to, Data envelopment analysis (DEA), Stochastic frontier analysis (SFA), Least-square econometric production models, Total factor productivity (TFP) indices.

Our search terms string for all the databases were: *(efficienc* OR productiv* OR performance OR inefficien*) AND ("data envelopment" OR DEA OR stochastic OR SFA OR parametric OR econometric* OR non-parametric OR nonparametric OR malmquist) AND (aged OR ageing OR aging OR "aged care" OR residential OR retirement OR "nursing home" or "long term care" OR "assisted living")*. Our search results as below.

	**Search strategy (1995 -2017; English only)**		**Medline**	**Econlit**	**Web of science**
#1	efficienc* OR productiv* OR performance OR inefficien*	Abstract	850,359	135,964	3,943,844
#2	"data envelopment" OR DEA OR stochastic OR SFA OR parametric OR econometric* OR non-parametric OR nonparametric OR malmquist	Abstract	67,570	49,136	418,570
#3	age* OR "aged care" OR residential OR retirement OR "nursing home" or "long term care" OR "assisted living"	Abstract	2,201,135	80,731	2,163,357
#4	#1 AND #2 AND #3	Abstract	**896**	**866**	**1860**

Appendix 3 - Detailed description of the included studies

No.	First author	Year	Country	Facility type, sample size, year	Efficiency measures	Estimation Methods	Inputs (I)	Outputs (O)	Other variables (Z)	No. of models
1	Anderson [11]	1999	USA	NH (653, 1995)	VRS, ITE	SFA (cost function, Bayesian, translog, 1 stage)	I1 = Total expense	O1 = Admissions	Z1 = For-profit status<br>Z2 = Chain	3
2	Anderson [12]	2003	USA	NH (487, 1996)	VRS, ITE	DEA (1 stage)	I1 = Residential costs<br>I2 = Overhead expense<br>I3 = Property expense<br>I4 = Other cost<br>I5 = Total operating cost<br>I6 = Ancillary services cost	O1 = Total bed days<br>O2 = Maximum bed days available<br>O3 = Utilisation rate (O1/ O2)		11
3	Bjorkgren [13]	2001	Finland	LTC unit (64, 1995)	CRS, ITE	DEA (production function, multiple regression, 2 stages)	I1 = FTE RNs<br>I2 = FTE LPNs<br>I3 = FTE aids<br>I4 = Beds	O1 = Case-mix adjusted resident days		2
4	Bjorkgren [14]	2004	Finland	LTC unit	VRS, ITE	DEA (production function, 1 stage)	I1 = FTE RNs<br>I2 = FTE LPNs<br>I3 = FTE aids<br>I4 = Beds	O1 = Case-mix resident days		3
5	Chang [15]	2013	Taiwan	NH (22; 2004-09)	CRS & VRS, ITE	DEA (truncated distribution, Tobit, 2 stages)	I1 =Number of employees<br>I2 = Floor area (m2)<br>I3 = Beds	O1 = Residents<br>O2 [QOC] = Falls<br>O3 [QOC] = Emergencies	Z1 = Licensed nurses<br>Z2 = Occupancy rate<br>Z3 = Government-expense NH<br>Z4 = Self-expense NH<br>Z5-9 = Year 2004-8	2

No.	First author	Year	Country	Facility type, sample size, year	Efficiency measures	Estimation Methods	Inputs (I)	Outputs (O)	Other variables (Z)	No. of models
6	Chattopadhyay [16]	1996	USA	NH (140; 1982-83)	CRS & VRS, OTE	DEA (2 stages)	I1 = Dietary staff hours<br>I2 = Housekeeping staff hours<br>I3 = Laundry staff hours<br>I4 = Nursing Directorhours<br>I5 = RN hours<br>I6 = LPN hours<br>I7 = Aides hours<br>I8 = Non-labour expenses	O1 = Medicare resident days<br>O2 = Medicaid resident days<br>O3 = Private resident days<br>O4 = Other resident days<br>O6 = ADL index (not claimed as quality)		1
7	Chen [17]	2004	USA	NH (4,635; 1994)	VRS, ITE	OLS (hybrid cost function, Tobit, 2-stages)	I1 = Wage	O1 = Medicare resident days<br>O2 = Medicaid resident days<br>O3 = Private resident days<br>O4 [QOL] = FTEs contribute to QOL<br>Q5 [QOC] = FTEs contribute to QOC<br>Q6 [QOL] = Involvement in organising groups for residents/families<br>Q7-10 [QOC] = Restrains; Catherisation; Drug error; Deficiencies (%)	Z1 = ADL index (not claimed as quality)<br>Z2-3 = Profit; Non-profit<br>Z4-5 = Hospital based; Chain affiliation<br>Z6 = HHI<br>Z7-8 = Metropolitan; Urban<br>Z9-16 = 8 geographic area dummies<br>Z17-19 = 3 dichotomous variables to indicate the measures of state Medicaid payment policy	1

No.	First author	Year	Country	Facility type, sample size, year	Efficiency measures	Estimation Methods	Inputs (I)	Outputs (O)	Other variables (Z)	No. of models
8	Crivelli [18]	2002	Switzerland	NH (886; 1998)	VRS, ITE	SFA (cost function, translog)	I1 = Price of labour<br>I2 = Price of capital	O1 = Total resident days	Z1 [QOC] = Average assistance time<br>Z2 = Average reimbursement<br>Z3 = Care persons/ resident ratio (not claimed as quality)<br>Z4 = No. of services provided<br>Z5 = Apartment NH<br>Z6 = Cantonal dummy variables<br>Comparative variables:<br>Z7-9 = Public; Private non-profit; Private for profit<br>Z10-14 = 5 types of regulatory settings	1
9	DeLellis [19]	2013	USA	NH (1,430; 2008)	VRS, ITE	DEA (linear programming modelling; 2 stages)	I1 = FTE RNs<br>I2 = FTE LPNs<br>I3 = FTE aids<br>I4 = FTE others<br>I5 = Beds	O1 = No. Medicare residents<br>O2 = No. Medicaid residents<br>O3 = No. Other residents	Comparative variables:<br>Z1-2 = Urban-Rural<br>Z3-4 = Chain-No chain<br>Z5-6 = Income <, > $34,000<br>Z7-8 = For-Not for profit<br>Z9-10 = HHI < and > average<br>Z11-12 = No. agencies in the county (≥, < 15)<br>Comparative variables [QOC]: Residents with<br>Z13 = Catheter	1

No.	First author	Year	Country	Facility type, sample size, year	Efficiency measures	Estimation Methods	Inputs (I)	Outputs (O)	Other variables (Z)	No. of models
									Z14-15 = Restrain: Total & Excluding physician order<br>Z16-17 = Pneumococcal and Influenzas vaccinations<br>Z18 = On pain management<br>Z19 = Pressure sores<br>Z20 = Bedfast<br>Z21 = Depression<br>Z22-23 = Incontinent of bladder-bowel<br>Z24 = Weight change<br>Facility:<br>Z25-26 = Acuity-ADL index<br>Z27 = Average No. ADL limitations	
10	Dervaux [20]	2006	France	NH (100; N/A)	CRS & VRS, orientation N/A	DEA (indirect output distance function, cost indirect revenue function)	I1 = FTE auxiliary personnel<br>I2 = Beds<br>I3 = Capital price<br>I4 = Labour price<br>I5 = Price for other charges (per day)	O1-6 = Case-mix resident days, by classification 1-6 (ADL & resource needs)		3

No.	First author	Year	Country	Facility type, sample size, year	Efficiency measures	Estimation Methods	Inputs (I)	Outputs (O)	Other variables (Z)	No. of models
11	Di Giorgio [21]	2015	Switzerland	NH (45; 2001-05)	VRS, ITE	SFA (cost function, True random effect models with and without Mundlak correction)	I1 = Price of labour<br>I2 = Price of capital<br>I3 = Price of material	O1 = Total resident days	Z1 [QOC] = ADL index<br>Z2 [QOC] = Nursing staff ratio (Ratio of No. of employed / guideline (optimal) nurses)<br>Z3-6 = Year 2002-05<br>Z7 = Institutional forms	4
12	Dormont [22]	2012	France	NH (1,171; 2007 - 740; 2003 & 2007)	VRS, ITE	SFA (translog cost function, maximum likelihood estimation, normal truncated, quantile, random effects, correlate random effects regressions, 2 stages)	I1 = Wages of nurses<br>I2 = Wage of nursing auxiliaries<br>I3 = Wage of non-nursing staff	O1 = Total resident days	Z1 = Ownership<br>Z2 = Urbanisation level<br>Z3 = No. years since last construction/ renovation)<br>Z4-9 = % residents in GIR groups 1-6 (ADL & resource needs)<br>Z10 = Receive Alzheimer residents<br>Z11 = Have reimbursement choice<br>Z12 = Have pharmacy<br>Z13 = Institutional form<br>Z14 = % social allowance<br>Z15 = GDP per capita<br>Z16 [QOC] = Staff/ Residents ratio<br>Z17 [QOC] = Non-nursing staff/ Nursing staff ratio	10

No.	First author	Year	Country	Facility type, sample size, year	Efficiency measures	Estimation Methods	Inputs (I)	Outputs (O)	Other variables (Z)	No. of models
13	Duffy [23]	2006	USA	LTC (69; N/A)	CRS, ITE	DEA (1 stage)	I1 = RN FTE / Resident days<br>I2 = LVN FTE / Resident days<br>I3 = Other FTE / Resident days<br>I4 = Dietary expense<br>I5 = Administrative expense<br>I6-7 = Professional and other staff salaries per resident day<br>I8 [QOC] = % non-ambulatory residents<br>I9 [QOC] = % not self-feeding	O1 = Total resident days<br>O2 [QOC] = % NO pressure ulcers		8
14	Dulai [24]	2016	USA	NH (761; 2009 -919; 2012)	VRS, ITE	SFA (hybrid translog cost function, truncated, 1 stage)	I1 = Price of RNs<br>I2 = Price of LPNs<br>I3 = Price of aids<br>I4 = Price of management	O1 = Total resident days<br>O2 = Discharges<br>O3 = Case-mix (minutes)<br>O4 [QOC] = Star rating for quality measures<br>Q5 [QOC] = Star rating from the health inspection	Z1 [QOC]= Average score of staffing ratings<br>Z2 = % Medicare residents<br>Z3 = % Medicaid residents<br>Z4 = For-profit status<br>Z5 = Chain<br>Z6 = Time trend	1
15	Dulai [25]	2017	USA	NH (338; 2009-2013)	VRS, ITE	DEA (Tobit, bootstrap, 2 stages,)	I1 = FTE RNs<br>I2 = FTE LPNs<br>I3 = FTE aids<br>I4 = FTE managementI5 = Beds	O1 = Total resident days<br>O2 = Discharges<br>O3 = Casemix (minutes)<br>O4 [QOC] = Average score of quality measures ratings	Z1 [QOC]= Average score of staffing ratings<br>Z2 = % Medicare residents<br>Z3 = % Medicaid residents<br>Z4 = For-profit status	1

No.	First author	Year	Country	Facility type, sample size, year	Efficiency measures	Estimation Methods	Inputs (I)	Outputs (O)	Other variables (Z)	No. of models
								O5 [QOC} = Average score of health inspection ratings	Z5 = Chain<br>Z6 = Time trend	
16	Farsi [26]	2004	Switzerland	NH (36; 1993-2001)	VRS, ITE	SFA (cost function, translog, random effects, 2 stages)	I1 = Price of labour<br>I2 = Price of capital	O1 = Total resident days	Z1 [QOC] = ADL index<br>Z2 [QOC] = Nursing staff ratio (Ratio of No. of employed / guideline (optimal) nurses)<br>Z3 = Linear time trend	2
17	Farsi [27]	2005	Switzerland	Non-profit NH (36; 1993-2001)	VRS, ITE	SFA (cost function, translog, fixed effects, random effects (GLS) with and without Mundlak formulation, pooled frontier, true random effects with and without Mundlak formulation)	I1 = Price of labour<br>I2 = Price of capital	O1 = Total resident days	Z1 [QOC] = ADL index<br>Z2 [QOC] = Nursing staff ratio (Ratio of No. of employed / guideline (optimal) nurses)<br>Z3 = Linear time trend	6

No.	First author	Year	Country	Facility type, sample size, year	Efficiency measures	Estimation Methods	Inputs (I)	Outputs (O)	Other variables (Z)	No. of models
18	Farsi [28]	2008	Switzerland	NH (356; 1998-2002)	VRS, ITE	SFA (cost function, pooled frontier model, random effect model using GLS method, true random effect model)	I1 = Price of labour<br>I2 = Price of capital	O1 = Total resident days	Z1 [QOC] = Average assistance time<br>Z2 = Average reimbursement<br>Z3 = Apartment NH<br>Z4 [QOC] = Care persons/ resident ratio (Dummy: >0.424: High quality facility)<br>Z5 = Linear time trend	3
19	Filippini [29]	2001	Switzerland	Non-profit NH (36; 1993-95)	VRS, ITE	Translog cost function	I1 = Price of labour<br>I2 = Price of capital	O1 = Total resident days	Z1 [QOC] = ADL index<br>Z2 [QOC] = Nursing staff ratio (Ratio of No. of employed / guideline (optimal) nurses)<br>Z3 = Apartment NH<br>Z4 = Time variable	1
20	Fried [30]	1998	USA	Nursing facilities (496; 1988)	CRS & VRS, ITE	DEA (cost approach, 2 stages)	I1 = Total expenses (payroll and not payroll)	O1 = Total resident days<br>O2 [QOC] = % non-medicaid resident days		1
21	Garavaglia [31]	2011	Italia	NH (40; 2005-07)	CRS, ITE	DEA (homogenous bootstrap, 2 stages, Tobit regression, Kruskull-Wallis test for hypothesis)	I1 = Health and nursing costs<br>I2 = Residential costs	O1 = Case-mix<br>O2 [QOC] = Extra nursing hours<br>O3 [QOC] = Residential charges	Z1 = Ownership<br>Z2 = Beds<br>Z3 = % lower severity	1

No.	First author	Year	Country	Facility type, sample size, year	Efficiency measures	Estimation Methods	Inputs (I)	Outputs (O)	Other variables (Z)	No. of models
22	Hsu [32]	2015	Canada	LTC (627; 1996-2011)	VRS, orientation N/A	Translog production function SFA; production function SFA, quantile regression; fixed effects model; GLE model	I1-5 = Hours of RNs, RPNs, therapists, aides, general staff hours<br>I6 = Care expense<br>I7 = Operational expense<br>I8 = Drug and medical equipment expense	O1 = Adjusted resident days	Z1-2 = Ownership (Municipal, non-profit)<br>Z3 = Chain<br>Z4 = Urban<br>Z5 = HHI<br>Z6-7 = Beds (lower and upper quartile)<br>Z8 [QOC] = Adjusted mortality rate<br>Z9 = Time trend	4
23	Knox [33]	1999	USA	NH (921; 1994)	DRS, profit orientation	Cobb-Douglas profit function, OLS, least trimmed squares	I1 = Price of labour (average LVN and Aid hourly wage)<br>I2 = Floor area<br>I3 = Occupancy rate	Profit function:<br>O2 = ADL index (as output price variable)	Z1 = Urban<br>Z2 = For-profit status<br>Z3 = Chain	1
24	Knox [34]	2003	USA	NH (1,017; 1994 - 983; 1998)	VRS, ITE, profit orientation	Modified reduced-form, translog cost- and profit-function regression techniques (both OLS and robust distance L one norm RDL1), 3 steps	I1 = Price of labour (average LVN and Aid hourly wage)<br>I2 = Floor area<br>I3 = Beds	Cost function:<br>O1 = Total resident days<br>Profit function:<br>O2 = ADL index (as output price variable)	Z1 = Urban<br>Z2 = For-profit status<br>Z3 = Chain<br>Z4 [QOC, not claimed QOL] = Quality rating<br>Z5 = Occupancy rate<br>Z6 = ADL index	6

No.	First author	Year	Country	Facility type, sample size, year	Efficiency measures	Estimation Methods	Inputs (I)	Outputs (O)	Other variables (Z)	No. of models
25	Knox [35]	2007	USA	NH (1,017; 1999-2002)	CRS, orientation N/A	SFA (production function Cobb-Douglas, half normal maximum likelihood estimator, quantile regression)	I1 = Beds<br>I2-7 =FTE hours of RNs, LVNs, Aids, other care staff, food staff	O1 = Total resident days	Z1 = For-profit status<br>Z2 = Year 1999	1
26	Knox [36]	2006	USA	Non-profit NH (143; 1994 - 138; 1998 - 161; 1999)	VRS, ITE, profit orientation	Modified reduced-form, translog cost- and profit-function regressiontech niques (both OLS and robust distance L one norm), 3 steps	I1 = Price of labour (average LVN and Aid hourly wage)<br>I2 = Beds	Cost function:<br>O1 = Total resident days<br>Profit function:<br>O2 = ADL index (as output price variable)	Z1 = Urban<br>Z2 = Ownership<br>Z3 = Chain<br>Z4 = Religious<br>Z5 = Occupancy rate<br>Z6 = ADL index<br>Z7-8 = Year 1998, 1999Z9? [QOC, not claimed QOL] = Quality rating (Dependent variable)	3
27	Laine [37]	2005a	Finland	LTC wards (122; 2001)	CRS, ITE	SFA (product function, truncated, 2 stages)	I1 = Beds<br>I2-4 = FTE RNs; LPNs; aides	O1 = Adjusted resident days	Z1 = Occupancy rate<br>Z2 = Facility type<br>Z3 = Ward specification<br>Z4 = Mean age<br>Z5 [QOC] = Pressure sores<br>Z6 [QOC] = % depression<br>Z7 [QOC] =	1

No.	First author	Year	Country	Facility type, sample size, year	Efficiency measures	Estimation Methods	Inputs (I)	Outputs (O)	Other variables (Z)	No. of models
									antipsychotic, anti-anxiety/hypnotic use	
28	Laine [38]	2005b	Finland	LTC ward (114; 2002)	CRS, ITE	DEA (Mann–Whitney test, 2 stages, correlation coefficients used to explore the association between quality and efficiency)	I1 = Beds<br>I2-4 = FTE RNs; LPNs; aides	O1 = Adjusted resident days	Z1 [QOC] = % RNs<br>Z2 [QOC] = % rooms with toilet<br>Z3 [QOC] = % single rooms<br>Z4-11 [QOC] = 7 ADL measures + % residents lack of training or range of motion<br>Z12-14 [QOC] = % pressure sores (new, low, high risk)<br>Z15-16 [QOC] = % catherisations (low, high risk)<br>Z17-19 [QOC] = % restrains, bestfast, weight change<br>Z20-21 [QOC] = % depression (with, without treatment)<br>Z22-26 [QOC] = antipsychotic, anti-anxiety/hypnotic use<br>Z27 [QOC] = % behavioural symptoms (total, low, high)<br>Z28 [QOC] = % cognitive impairment	1

No.	First author	Year	Country	Facility type, sample size, year	Efficiency measures	Estimation Methods	Inputs (I)	Outputs (O)	Other variables (Z)	No. of models
									Z29 [QOC] = % >= 9 medications<br>Z30-34 [QOC] = % bowel or bladder continence Z35 [QOC] = % UTI<br>Z36-38 [QOC] = % injuries, falls, fractures	
29	Laine [39]	2005c	Finland	LTC wards (113; 2001-2002)	VRS, ITE	SFA (cost function, translog, truncated, 2 stages)	I1 = Average wage rate	O1 = Adjusted resident days<br>O2 [QOC] = % pressure sores<br>O2 [QOC] = % depression without treatment	Z1 = Facility type<br>Z2 [QOC]= % restrains<br>Z3 [QOC] = % depressants and hypnotic use	1
30	Lin [40]	2017	Taiwan	Senior care facilities (91; 2011)	CRS, ITE	CCR, slacks-based measure, and epsilon-based measure DEA models, metafriontier efficiency analysis, least square regression, applied chain rules to regression; Production function, 2 stages	I1 = Nursing personnel<br>I2 = Non-nursing personnel<br>I3 = Floor area	O1 = Residents	Z1 [QOC] = Facility rating (A or B)<br>Z2 = Facility type (General senior care or nursing home)<br>Z3 = Occupancy rate	3

No.	First author	Year	Country	Facility type, sample size, year	Efficiency measures	Estimation Methods	Inputs (I)	Outputs (O)	Other variables (Z)	No. of models
31	Min [41]	2016	USA	NH (2267; 2010)	CRS, ITE	DEA (linear regression, 3-level modelling (NH, county, state), 2 stages)	I1 = FTE hours of RNs (per resident day)<br>I2 = FTE hours of LPNs (per resident day)<br>I3 = FTE hours of Aids (per resident day)	O1 [QOC] = % pain<br>O2 [QOC] = % ADL decline<br>O3 [QOC] = % pressure sores<br>O4 [QOC] = % restraints<br>O5 [QOC] = % UTI<br>O6 [QOC] = % falls	Z1 = For-profit status<br>Z2 = Chain<br>Z3 = % Medicare residents<br>Z4 = % Medicaid residents<br>Z5 = Beds<br>Z6 = Occupancy rate<br>Z7 = Acuity index	2
32	Ni Nuasa [42]	2016	Ireland	NH (152; 2008-09)	CRS & VRS, ITE	DEA (bootstrap, 2 stages)	I1 = Medical personnel<br>I2 = Non-medical personnel<br>I3 = Beds	O1 = Total resident days	Z1 = Ownership<br>Z2 = Location<br>Z3 [QOC] = Qualification of nurse<br>Z4-6 = Beds (0-49; 50-99; >=100)<br>Z7 = Casemix (age)	3
33	Ozcan [43]	1998	USA	Skilled nursing facilities (324, 1990-91)	VRS, ITE	DEA (Slack analysis, Post hoc logistic regression, 2 stages)	I1 = Beds<br>I2 = FTEs<br>I3 = Operational expenses	O1 = Medicare & Medicaid resident days<br>O2 = Private resident days	Z1 = % Medicare residents<br>Z2 = % Medicaid residents<br>Z3 = Occupation rate<br>Z4 = Region<br>Z5 = % of population ≥ 84 years	1

No.	First author	Year	Country	Facility type, sample size, year	Efficiency measures	Estimation Methods	Inputs (I)	Outputs (O)	Other variables (Z)	No. of models
34	Rosko [44]	1995	USA	NH (461; 1987)	CRS, profit orientation	DEA (X-efficiency theory, Tobit, 2 stages)	I1-5 = FTE RNs, LPNs, Aides, Rehab, Other	O1 = Skilled nursing facility days<br>O2 = Intermediate care facility days	Z1 = For-profit<br>Z2 = HHI<br>Z3 = County occupancy rate<br>Z4 = Per capita personal income<br>Z5 = Wage index<br>Z6 = % Medicare residents<br>Z7 = % Medicaid residents<br>Z8-9 = Beds, Beds squared<br>Z10 = Occupancy rate<br>Z11 = Resident case-mix index<br>Z12 = % residents > 85 years<br>Z13 = % Confused<br>Z14 = Independent living capacity<br>Z15 = Discharge rate<br>Z16-18 [QOC] = % pressure sores, restraint, catheter	1
35	Shimshak [45]	2007	USA	NH (38; 2003)	VRS, ITE	DEA (1 stage)	I1 = FTEs	O1 = Residents<br>O2-6 = Case-mix severity (assistance with bathing, dressing, transferring, toileting, and eating)<br>O7-9 [QOC] = Residents without pressure sores,		2

No.	First author	Year	Country	Facility type, sample size, year	Efficiency measures	Estimation Methods	Inputs (I)	Outputs (O)	Other variables (Z)	No. of models
								restraints, and catheterisations		
36	Shimshak [46]	2009	USA	NH (38; 2003)	CRS, ITE	DEA (1 stage)	I1-6 = FTE RNs, LPNs, Aides, ancillary non-nursing professional staff, ancillary non-nursing nonprofessional staff, and administrative staff	O1 = Residents<br>O2-6 = Case-mix severity (assistance with bathing, dressing, transferring, toileting, and eating)<br>O7-9 [QOC] = Residents without pressure sores, restraints, and catheterisations		7
37	Shimshak [47]	2010	USA	NH (91; 2003)	CRS, ITE	DEA (1 stage)	I1-6 = FTE RNs, LPNs, Aides, ancillary non-nursing professional staff, ancillary non-nursing non-professional staff, and administrative staff	O1 = Residents<br>O2-6 = Case-mix severity (assistance with bathing, dressing, transferring, toileting, and eating)<br>O7-9 [QOC] = Residents without pressure sores, restraints, and catheterisations		4
38	Wang [48]	2005	Taiwan	LTC (53; 1995)	CRS & VRS, ITE	DEA (OLS, 2 stages)	I1 = Beds<br>I2-5 = No. of doctors, physical therapists, pharmacists, dietitians<br>I6 = Non-medical staff<br>I7 = Nursing staff	O1 = Residents<br>O2 [QOC] = Administrative service performance<br>O3 [QOC] = Life care performance<br>O4 [QOC] = Health care performance<br>O5 [QOC] = Accident rate	Z1 = Ownership<br>Z2 = Municipal supervision<br>Z3 = Beds<br>Z4 = Occupancy rate<br>Z5 = Facility type<br>Z6 = HHI	8

No.	First author	Year	Country	Facility type, sample size, year	Efficiency measures	Estimation Methods	Inputs (I)	Outputs (O)	Other variables (Z)	No. of models
39	Zhang [49]	2008	USA	NH (8,361; 1997-2003)	VRS, ITE	DEA (bootstrap, truncated, 2 stages)	I1 = General service expense<br>I2 = Routine services expense<br>I3 = Ancillary services expense	O1-3 = Resident days (Skilled nursing, intermediate nursing and other long-term care)<br>O4 [QOC] = Deficiencies	Z1 = Ownership<br>Z2 = Profit status<br>Z3 = Chain<br>Z4-5 = % Medicare, Medicaid residents<br>Z6 = Beds<br>Z7 = Occupancy rate<br>Z8 = RN/total nursing staff<br>Z9 = RN hours/resident day<br>Z10 = HHI<br>Z11 = Medicaid reimbursement<br>Z12-14 = Medicare police changes	3

ADL = Activity of daily living; CCR = Charnes, Cooper, and Rhodes DEA model; CRS = constant returns to scale; DEA = data envelopment analysis; DRS = decreasing returns to scale; FTE = full-time equivalent; GLS = generalised least squares;GDP = gross domestic product; HHI = Herfindahl-Hirschman Index; ITE = input-oriented technical efficacy; LTC = long-term care; LPN = licensed practical nurse ; LVN= licensed vocational nurse; N/A = not available; NH = nursing home; No. = number of; OLS = ordinary least squares; OTE = output-oriented technical efficacy; QOC = quality of care; QOL = Quality of life; RN = registered nurse; RTS = returns to scale; SFA = stochastic frontier analysis; UTI = urinary tract infection; VRS = variable returns to scale.

References

1. O'Donnell CJ. Productivity and efficiency analysis: an economic approach to measuring and explaining managerial performance / Christopher J. O'Donnell. Singapore: Springer; 2018.

2. Cooper WW, Seiford LM, Zhu J eds. Handbook on Data Envelopment Analysis [Internet]. Second edition. International Series in Operations Research and Management Science, vol. 164. New York and Heidelberg: Springer; 2011. xxv. Available from: http://search.ebscohost.com/login.aspx?direct=true&db=ecn&AN=1300017&site=ehost-live

3. Coelli T, Rao DSP, O'Donnell CJ, Battese GE. An Introduction to Efficiency and Productivity Analysis. Boston, MA : Springer US : Imprint: Springer; 2005.

4. Farrell MJ. The Measurement of Productive Efficiency. Journal of the Royal Statistical Society Series A (General). 1957;120(3):253–90.

5. Charnes A, Cooper WW, Rhodes E. Measuring the efficiency of decision making units. European Journal of Operational Research. 1978 Nov 1;2(6):429–44.

6. Banker RD, Charnes A, Cooper WW. Some Models for Estimating Technical and Scale Inefficiencies in Data Envelopment Analysis. Management Science. 1984;30(9):1078–1092.

7. Färe R, Grosskopf S, Logan J. The relative efficiency of Illinois electric utilities. Resources and Energy. 1983;5(4):349–367.

8. Aigner D, Lovell CAK, Schmidt P. Formulation and estimation of stochastic frontier production function models. Journal of Econometrics. 1977 Jul 1;6(1):21–37.

9. Meeusen W, van Den Broeck J. Efficiency Estimation from Cobb-Douglas Production Functions with Composed Error. International Economic Review. 1977;18(2):435.

10. Battese GE, Corra GS. ESTIMATION OF A PRODUCTION FRONTIER MODEL: WITH APPLICATION TO THE PASTORAL ZONE OF EASTERN AUSTRALIA. Australian Journal of Agricultural Economics. 1977;21(3):169–179.

11. Anderson RI, Lewis D, Webb JR. The efficiency of nursing home chains and the implications of non-profit status. Journal of Real Estate Portfolio Management; Boston. 1999;5(3):235–45.

12. Anderson RI, Weeks HS, Hobbs BK, Webb JR. Nursing home quality, chain affiliation, profit status and performance. The Journal of Real Estate Research; Sacramento. 2003 Mar;25(1):43–60.

13. Bjorkgren MA, Hakkinen U, Linna M. Measuring Efficiency of Long-Term Care Units in Finland. Health Care Management Science. 2001 Sep;4(3):193–200.

14. Björkgren MA, Fries BE, Häkkinen U, Brommels M. Case-mix adjustment and efficiency measurement. Scandinavian Journal Of Public Health. 2004;32(6):464–71.

15. Chang S-J, Cheng M-A. The impact of nursing quality on nursing home efficiency: evidence from Taiwan. Review of Accounting Finance. 2013 Oct 28;12(4):369–86.

16. Chattopadhyay S, Ray SC. Technical, scale, and size efficiency in nursing home care: a nonparametric analysis of Connecticut homes. Health Economics. 1996 Aug 7;5(4):363–73.

17. Chen LW, Shea DG. The economies of scale for nursing home care. Med Care Res Rev. 2004 Mar;61(1):38–63.

18. Crivelli L, Filippini M, Lunati D. Regulation, ownership and efficiency in the Swiss nursing home industry. International Journal Of Health Care Finance And Economics. 2002 Jun;2(2):79–97.

19. DeLellis NO, Ozcan YA. Quality outcomes among efficient and inefficient nursing homes: a national study. Health Care Management Review. 2013 Jun 4;38(2):156–65.

20. Dervaux B, Leleu H, Nogues H, Valdmanis V. Assessing French nursing home efficiency: An indirect approach via budget-constrained DEA models. Socio-Economic Planning Sciences. 2006 Mar;40(1):70–91.

21. Di Giorgio L, Filippini M, Masiero G. Structural and managerial cost differences in nonprofit nursing homes. Econ Model. 2015 Dec;51:289–98.

22. Dormont B, Martin C. Quality of service and cost-efficiency of French nursing homes. In 2012. p. 21.

23. Duffy JAM, Fitzsimmons JA, Jain N. Identifying and studying “best-performing” services. Benchmarking. 2006;13(3):232–51.

24. Dulal R. Cost efficiency of nursing homes: do five-star quality ratings matter? Health Care Management Science. 2016 Jan 29;

25. Dulal R. Technical efficiency of nursing homes: do five-star quality ratings matter? Health Care Management Science. 2017 Feb 28;

26. Farsi M, Filippini M. An Empirical Analysis of Cost Efficiency in Non-profit and Public Nursing Homes. Annals of Public and Cooperative Economics. 2004 Sep;75(3):339–65.

27. Farsi M, Filippini M, Kuenzle M. Unobserved heterogeneity in stochastic cost frontier models: an application to Swiss nursing homes. Applied Economics. 2005 Oct 10;37(18):2127–41.

28. Farsi M, Filippini M, Lunati D. Economies of Scale and Efficiency Measurement in Switzerland’s Nursing Homes. Schweizerische Zeitschrift fur Volkswirtschaft und Statistik/Swiss Journal of Economics and Statistics. 2008 Sep;144(3):359–78.

29. Filippini M. Economies of scale in the Swiss nursing home industry. Applied Economics Letters. 2001 Jan 1;8(1):43–6.

30. Fried HO, Schmidt SS, Yaisawarng S. Productive, Scale And Scope Efficiencies In U.S. Hospital-Based Nursing Homes. INFOR: Information Systems and Operational Research. 1998 Aug;36(3):103–19.

31. Garavaglia G, Lettieri E, Agasisti T, Lopez S. Efficiency and Quality of Care in Nursing Homes: An Italian Case Study. Health Care Management Science. 2011 Mar;14(1):22–35.

32. Hsu AT-M. An Investigation of Approaches to Performance Measurement: Applications to Long-Term Care in Ontario [Internet] [Ph.D.]. [Ann Arbor]: University of Toronto (Canada); 2015. Available from: http://search.proquest.com.libraryproxy.griffith.edu.au/docview/1758252389?accountid=14543

33. Knox KJ, Blankmeyer EC, Stutzman JR. Relative economic efficiency in Texas nursing facilities: A profit function analysis. Journal of Economics and Finance. 1999 Sep;23(3):199–213.

34. Knox KJ, Blankmeyer EC, Stutzman JR. Organizational Efficiency and Quality in Texas Nursing Facilities. 2003;14.

35. Knox KJ, Blankmeyer EC, Stutzman JR. Technical Efficiency in Texas Nursing Facilities: A Stochastic Production Frontier Approach. Journal of Economics and Finance. 2007;31(1):75–86.

36. Knox KJ, Blankmeyer EC, Stutzman JR. Comparative Performance and Quality Among Nonprofit Nursing Facilities in Texas. Nonprofit and Voluntary Sector Quarterly. 2006 Dec 1;35(4):631–67.

37. Laine J, Linna M, Hakkinen U, Noro A. Measuring the Productive Efficiency and Clinical Quality of Institutional Long-Term Care for the Elderly. Health Economics. 2005;14(3):245–56.

38. Laine J, Finne-Soveri UH, Björkgren M, Linna M, Noro A, Häkkinen U. The association between quality of care and technical efficiency in long-term care. International Journal For Quality In Health Care: Journal Of The International Society For Quality In Health Care. 2005 Jun;17(3):259–67.

39. Laine J, Linna M, Noro A, Hakkinen U. The Cost Efficiency and Clinical Quality of Institutional Long-Term Care for the Elderly. Health Care Management Science. 2005 May;8(2):149–56.

40. Lin J-R, Chen C-Y, Peng T-K. Study of the Relevance of the Quality of Care, Operating Efficiency and Inefficient Quality Competition of Senior Care Facilities. Int J Environ Res Public Health [Internet]. 2017 Sep [cited 2018 Oct 2];14(9). Available from: https://www.ncbi.nlm.nih.gov/pmc/articles/PMC5615584/

41. Min A, Park CG, Scott LD. Evaluating Technical Efficiency of Nursing Care Using Data Envelopment Analysis and Multilevel Modeling. Western Journal of Nursing Research. 2016 Nov;38(11):1489–508.

42. Ni Luasa S, Dineen D, Zieba M. Technical and scale efficiency in public and private Irish nursing homes – a bootstrap DEA approach. Health Care Management Science [Internet]. 2016 Oct 27 [cited 2018 Jul 18]; Available from: http://link.springer.com/10.1007/s10729-016-9389-8

43. Ozcan YA, Wogen SE, Mau LW. Efficiency Evaluation of Skilled Nursing Facilities. 1998;14.

44. Rosko MDP, Chilingerian JAP, Zinn JSP, Aaronson WEP. The Effects of Ownership, Operating Environment, and Strategic Choices on Nursing Home Efficiency. Medical Care. 1995 Oct;33(10):1001–21.

45. Shimshak DG, Lenard ML. A Two-Model Approach to Measuring Operating and Quality Efficiency with DEA. INFOR: Information Systems and Operational Research. 2007 Aug;45(3):143–51.

46. Shimshak D, Lenard M, Klimberg R. Incorporating quality into data envelopment analysis of nursing home performance: A case study☆. Omega. 2009 Jun;37(3):672–85.

47. Shimshak DG. Managing Nursing Home Quality Using Dea with Weight Restrictions. In: Lawrence KD, Kleinman G, editors. Applications in Multicriteria Decision Making, Data Envelopment Analysis, and Finance. Bingley: Emerald Group Publishing Limited; 2010. p. 199–216.

48. Wang YH, Chou LF. The Efficiency of Nursing Homes in Taiwan: An Empirical Study Using Data Envelopment Analysis. 輔仁管理評論. 2005 Jan;12(1):167–94.

49. Zhang NJ, Unruh L, Wan TTH. Has the Medicare prospective payment system led to increased nursing home efficiency? Health Serv Res. 2008 Jun;43(3):1043–61.
